# 7 Alcohol Use Acuity and Burn Mortality: A Ten-Year Retrospective Analysis

**DOI:** 10.1093/jbcr/irae036.007

**Published:** 2024-04-17

**Authors:** Jamie L Hollowell, Ashley Levine, Benjamin Bodek, Chris B Agala, Eli Maxwell, Lori Chrisco, Robert W Matthews, Booker King, Felicia Williams

**Affiliations:** University of North Carolina, Chapel Hill, NC; University of North Carolina, Durham, NC; University of North Carolina Chapel Hill - School of Medicine, Surgery dept, Chapel Hill, NC; University of North Chapel Hill Jaycee Burn Center, Chapel Hill, North Carolina; University of North Carolina, Chapel Hill, NC; University of North Carolina, Durham, NC; University of North Carolina Chapel Hill - School of Medicine, Surgery dept, Chapel Hill, NC; University of North Chapel Hill Jaycee Burn Center, Chapel Hill, North Carolina; University of North Carolina, Chapel Hill, NC; University of North Carolina, Durham, NC; University of North Carolina Chapel Hill - School of Medicine, Surgery dept, Chapel Hill, NC; University of North Chapel Hill Jaycee Burn Center, Chapel Hill, North Carolina; University of North Carolina, Chapel Hill, NC; University of North Carolina, Durham, NC; University of North Carolina Chapel Hill - School of Medicine, Surgery dept, Chapel Hill, NC; University of North Chapel Hill Jaycee Burn Center, Chapel Hill, North Carolina; University of North Carolina, Chapel Hill, NC; University of North Carolina, Durham, NC; University of North Carolina Chapel Hill - School of Medicine, Surgery dept, Chapel Hill, NC; University of North Chapel Hill Jaycee Burn Center, Chapel Hill, North Carolina; University of North Carolina, Chapel Hill, NC; University of North Carolina, Durham, NC; University of North Carolina Chapel Hill - School of Medicine, Surgery dept, Chapel Hill, NC; University of North Chapel Hill Jaycee Burn Center, Chapel Hill, North Carolina; University of North Carolina, Chapel Hill, NC; University of North Carolina, Durham, NC; University of North Carolina Chapel Hill - School of Medicine, Surgery dept, Chapel Hill, NC; University of North Chapel Hill Jaycee Burn Center, Chapel Hill, North Carolina; University of North Carolina, Chapel Hill, NC; University of North Carolina, Durham, NC; University of North Carolina Chapel Hill - School of Medicine, Surgery dept, Chapel Hill, NC; University of North Chapel Hill Jaycee Burn Center, Chapel Hill, North Carolina; University of North Carolina, Chapel Hill, NC; University of North Carolina, Durham, NC; University of North Carolina Chapel Hill - School of Medicine, Surgery dept, Chapel Hill, NC; University of North Chapel Hill Jaycee Burn Center, Chapel Hill, North Carolina

## Abstract

**Introduction:**

The high incidence of alcohol consumption nationally is linked to increased susceptibility for burn injuries in both cases of acute intoxication as well as chronic misuse. While detection of elevated blood ETOH levels on admission intuitively does not always reflect a patient’s history of use, it does introduce bias and can influence clinical decision-making. Understanding these inherent biases at play, our institution through a retrospective analysis, sought to investigate the differing effects a positive blood alcohol level and true chronic alcohol use disorder had on overall mortality within our burn center.

**Methods:**

We conducted a retrospective analysis utilizing data from the Institutional Burn Center Registry, which was linked to clinical and administrative records. Our study included all adult patients admitted to the Burn Center between January 1, 2012, and December 31, 2022. We collected and analyzed various parameters, including patient demographics, length of hospital stay (LOS), comorbid conditions, and mortality outcomes. Using logistic regression modeling we evaluated the association between burn mortality and ETOH.

**Results:**

Over the ten-year study period, a total of 9,955 adult patients were admitted to our burn center. 6,943 tested negative and 541 patients tested positive for blood ETOH on admission. The remaining 2,471 had no test. 567 were determined to have a diagnosis of alcohol use disorder. A positive for ETOH did not have significant impact on mortality (p=0.76) while history of chronic ETOH use had a nearly three-fold increase in odds of mortality (OR 2.58, CI 1.52-4.24, p=0.000) when accounting for factors present in traditional predictive models such as burn size, age, and inhalational injury.

**Conclusions:**

Chronic alcoholism has a greater association with mortality than acute alcohol intoxication at our institution. Further, clinicians should exercise caution when presuming alcohol use disorder solely based on a positive test. This highlights the necessity of accurate medical history taking when estimating and counseling patients on mortality risk.

**Applicability of Research to Practice:**

This emphasizes the pressing need for research into the effects of multiple comorbidities and social determinants on mortality among burn patients. The insights gained have practical implications for refining clinical approaches and optimizing care strategies for this vulnerable patient population.

